# The Impact of Candle Burning During All Saints’ Day Ceremonies on Ambient Alkyl-Substituted Benzene Concentrations

**DOI:** 10.1007/s00128-013-1104-6

**Published:** 2013-09-20

**Authors:** Tomasz Olszowski, Andrzej Kłos

**Affiliations:** 1 Department of Thermal Engineering and Industrial Facilities, Faculty of Mechanical Engineering, Opole University of Technology, Mikołajczyka 5, 45-271 Opole, Poland; 2 Chair of Biotechnology and Molecular Biology, Opole University, kard. B. Kominka 4, 45-032 Opole, Poland

**Keywords:** Ambient air, BTEXS, Passive samplers, Cemetery

## Abstract

Research findings concerning benzene, toluene, ethylobenzene, meta-, para- and ortho-xylene as well as styrene (BTEXS) emission at public cemeteries during All Saints’ Day are presented here. Tests were carried out at town-located cemeteries in Opole and Grodków (southern Poland) and, as a benchmark, at the centres of those same towns. The purpose of the study was to estimate BTEXS emissions caused by the candle burning and, equally important to examine, whether emissions generated by the tested sources were similar to the BTEXS emissions generated by road transport. During the festive period, significant increases in benzene concentrations, by 200 % and 144 %, were noted at the cemeteries in Opole and Grodków, as well as in toluene, by 366 % and 342 %, respectively. Styrene concentrations also increased. It was demonstrated that the ratio of toluene to benzene concentrations from emissions caused by the burning candles are comparable to the ratio established for transportation emissions.

Light aromatic hydrocarbons (BTEXS) are classified as volatile organic compounds (VOC). Due to their toxic effect, the permissible ambient air concentration of some of these compounds is legally regulated (European Commission 2008). Benzene is the most toxic and has the greatest effect on human health. In accordance with the decision of the International Agency for Research on Cancer (IARC) – benzene is classified in Group 1 and is therefore considered to be a highly carcinogenic compound (IARC 2004). BTEXS have also been associated with various effects on health, including carcinogenic and/or mutagenic effects, which, apart from benzene, are also ascribed to ethylbenzene and styrene (Kyle et al. 2001; OEHHA 2009). Additionally, compounds in the BTEXS group may have an adverse effect on the nervous, respiratory and circulatory systems (Chen et al. 2008; OEHHA 2009). Monocyclic carbohydrates, apart from their toxic properties, are characterised by a large potential for creating tropospheric ozone (ground level ozone) and certain ones (toluene, ethylbenzene, xylenes) also participate in formation of the secondary organic aerosols that are harmful to human beings and ecosystems (Griffin et al. 1997; Rappengluck et al. 1998; Atkinson 2000; Khoder 2007).

Locally, in industrial and urban areas in particular, BTEXS concentrations in the environment largely depend on anthropogenic sources (Fernandez-Villarrenaga et al. 2004; Pyta and Zajusz 2010). In accordance with ESIG’s (European Solvents Industry Group, Brussels) data, the main anthropogenic sources of BTEXS are as follows: transport (35 %), solvent production and usage (24 %), other manufacturing processes (7 %) and energy incineration (6 %) (Caselli et al. 2010). Other tests indicate that BTEXS are found mainly in petroleum products (Chen et al. 2008). Since according to many authors, transport emissions are the major source of VOC in the city environment (Singh et al. 1992; Fernandes et al. 2002; Buczynska et al. 2009), they used to be a frequent object of study. Others (Mohamed et al. 2002; Pankow et al. 2003; Ohura et al. 2006; Hinwood et al. 2007; Jia et al. 2008) have also followed BTEXS air concentrations in the urban and industrial areas. Assessment of concentrations (including BTEXS) and the scale of the health hazard in closed spaces (Esplugues et al. 2010) as well as in workplaces (Weisel et al. 2005; Lee et al. 2006) has been also examined. Studies proved that VOC concentrations indoors very often exceeded those in the air outside (Breysse et al. 2004; Wallace 1991; Esplugues et al. 2010). What is seldom recognised as a source of BTEXS emission is burning candles at home and while celebrating various ceremonies, e.g. in commemorating someone who has passed away. While burning, candles emit quite significant amounts of soot, close to 0.06 μm (Makles and Pośniak 2003). That soot absorbs various chemical compounds, including VOCs (Lau et al. 1997). It is supposed that the combustion products emitted while burning candles, cemetery candles, etc., are similar in their composition to fumes emitted by diesel engines (Makles and Pośniak 2003). Candles for household and outdoor use (cemetery candles) are generally produced of paraffin, stearin or a mixture, rarely of beeswax or hardened vegetable oils. Burning paraffin candles appears to be the main hydrocarbon initiator, while burning stearin candles results in 50 % lower concentrations. In terms of toxicity, natural wax candles are the least toxic (Lau et al. 1997; Rezaei et al. 2002a, b; Pagels et al. 2009).

In many countries, e.g. in Poland, Austria, Croatia and Romania, there is a strong tradition of lighting candles at the graves of relatives in order to honour those who are gone. All Saints’ Day is celebrated in Poland on November 1st and on that day cemeteries are crowded with visitors. The same situation occurs few days before the festival and a few days after. Quite often at a tombstone, within a 2 m^2^ space, 50–100 cemetery candles are burning. It is estimated that on All Saints’ Day 2004 in Poland, 92 % of Poles (i.e. 33 million people) visited cemeteries, and the average period of stay was up to 2 h (TNS OBOP 2004). Moreover, cemetery candles are often made of non-refined paraffin, and their combustion products are characterised by increased toxicity (Makles and Posniak 2003).

So far, no one has studied the problem of an episode of increased concentrations of BTEXS in cemeteries on All Saints’ Day. Furthermore, no one has dealt with the issue of the environmental effects of pollution emitted by a large number of cemetery candles burning in the open spaces of public utility facilities all at the same time; thus, one should examine the undertaken research subject in that context.

The aim of the study was a quantitative analysis of BTEXS emitted during intense burning of candles in cemeteries. It has been hypothesized that the emission of BTEXS from candles and grave lights has an impact on the level of pollutants around cemeteries. The composition of this emission is comparable to emissions from transport and communications. The relative growth of BTEXS emissions and the possibility of transgressing acceptable benzene concentrations were taken into account.

## Materials and Methods

**Fig. 1:**
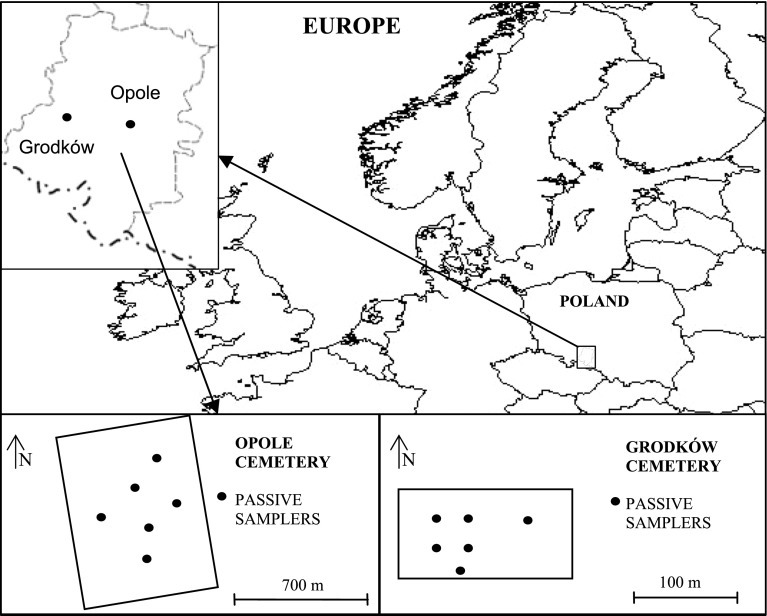
Map of measuring site locations

The tests were carried out at the municipal cemetery in Opole, the capital of Opole District (126,000 inhabitants) and in Grodków (9,000 inhabitants) as well as in the city centres, for the sake of comparison, in order to estimate the contribution of each city’s emissions to the level of emission at the cemeteries. The cemetery in Opole is located near the western administrative border of the city, 3 km from the city centre. Within an area of 0.3 km^2^ over 43,000 graves are located. The cemetery in Grodków is located 0.5 km from the town centre; it includes 1,200 graves and occupies an area of 0.01 km^2^. The locations of the spots where tests were carried out are presented in Fig. 1.

Modified Amaya badge-type permeation passive samplers (producer: Propagator, Kraków, Poland) were used for the assessment of BTEXS: benzene (B), toluene (T), ethylbenzene (E), meta-xylene (mX), para-xylene (pX), ortho-xylene (oX) and styrene (S) concentrations in the ambient air. This type of passive sampler has been used many times before, and their effectiveness has been verified (Smith et al. 2007; Ramadan 2010; Baltrenas et al. 2011). In Poland they are used by public entities responsible for monitoring ambient air pollution (GIOŚ 2003). A passive sampler with a 25 mm inner diameter and 10 mm depth is made of polyethylene. The volume of the activated carbon bed amounts to 0.85 cm^3^. The carbon bed is covered by a stainless steel net 23 mm in diameter (mesh diameter: 0.2 mm) and by a thrust collar with a microporous partition made of polypropylene unwoven fabric. The method consists of adsorption of the analytes into the activated carbon. Infiltration of BTEXS into the sampler’s interior occurs as a result of free diffusion. The samplers were exposed in the period between 21st and 27th October 2011 (first period) and between 28th October and 3rd November 2011 (second period).

The purpose of the exposure during the first testing period was to determine BTEXS concentrations in air for a period of time when increased cemetery candle burning is not observed, and it was assumed as a reference value. Twenty samples were exposed simultaneously during the first and second periods (6 pieces at each of the cemeteries in Opole and Grodków and 4 pieces in each city centre). Air sampling spots at the cemeteries were selected randomly, taking into consideration the maximum limitation of third party access. In the city centres, the samplers were put up at the main arteries. Samplers were exposed at a height 1.5–1.6 m above ground level, i.e. at the average height at which a human body is aspirating the air. In order to estimate the greatest intensity of emission at the cemeteries on November 1 and 2, two samplers were additionally exposed at each cemetery for 40 h (third period). In total, 44 samplers were exposed. In order to determine initial concentrations of BTEXS in the samplers, another 3 were left hermetically sealed in the laboratory (blanks).

Following the period of air sampling, BTEXS were extracted with carbon disulfide and, after evaporation, subjected to quantitative analysis by gas chromatography with a FID detector (Carlo Ebra). The external standard method was used for calibration. The effectiveness of this method of analysis in terms of volume has been confirmed by (Begerow et al. 1996; Propagator 2002; Tuduri et al. 2012). Desorption, chromatographic analysis and calibration, as well as calculation procedures were carried out as instructed (Begerow et al. 1996; Kalina 2002). BTEXS concentrations in air were calculated in accordance with the following formula:

1 $$ c_{i} = \, 1.44 \cdot m_{i} /P_{i} \cdot t $$

where: *c*_i_ – concentration of substance *i* in the air (μg m^−3^), *m*_i_ – mass of substance *i* determined in the sampler, minus mass of substance *i* in the blank specimen (μg), *t* – exposure time (min) and *p* – empirical conversion factor, characteristic for substance *i* (taken from tables), defined as the mass of substance *i* determined in the sampler after 24-h exposure of the sampler to the air containing 100 μg of substance *i* in 1 m^3^ of air, μg_(substance i)_/100 μg_(substance i)_/(m^3^ 24 h) (Kalina 2002).

Parameters concerning the applied method for determining BTEXS: method detection limit (*MDL*) and method quantification limit (*MQL*), precision (*RSD*), method accuracy (*MA*) as well as extended measurement uncertainty (*U*) are presented in Table 1. *MDL* values were determined on the basis of sampling for 40 and 168 h. These parameters were determined on the basis of the average results of tests of the composition of air being polluted from various BTEXS emitters (Instructions – Propagator, Kraków, Poland).

**Table 1:** Analytical parameters of the passive method for BTEXS determination in atmospheric air, n = 205 (40)

BTEXS	*MDL* ^a^ (μg m^−3^)	*MQL* (μg m^−3^)	*RSD* (%)	*MA* (%)	*U*
B	0.25 (0.7)	1.0	6.0	12	17
T	0.25 (0.7)	1.0	7.0	15	20
E	0.30 (0.9)	1.2	10	20	29
mX + pX	0.37 (1.0)	1.5	10	20	29
o-X	0.32 (1.0)	1.3	10	20	29
S	0.36 (1.0)	1.5	12	25	35

Instructions – propagator, Kraków, Poland

^a^In the bracket *MDL* values for 40 h exposition

Atmospheric conditions were also monitored. Average temperature during the exposure was 9.8 and 10.1°C in Opole and Grodków, respectively. The wind, at an average speed of 2 m s^−1^, was blowing from the Southwest in Opole and from the South in Grodków. The air pressure was stable and showed 999–1,008 hPa. No precipitation occurred. Atmospheric conditions (in particular speed and wind direction) occurring on the days of sampler exposure significantly limited the quantity of city emissions at the cemeteries.

Since the distribution of our measurement results was unknown, the Wilcoxon test was used to compare the concentrations. This non-parametric statistical test was used to assess whether one of two samples of independent observations (the results of concentration measurements) tends to have higher values than the other. The bilateral confidence level was considered in testing at the critical *p* value equal to 0.05. The Wilcoxon test was used to estimate differences in BTEXS concentrations in the first and second period of testing at the cemeteries.

## Results and Discussion

BTEXS concentration values after seven days of air sampling at the cemeteries, calculated on the basis of Eq. 1, are presented in Table 2. Average, median, minimum and maximum values as well as SD are given in Table 2.

**Table 2:** BTEXS concentrations in the air (μg m^−3^) at the cemeteries during the first (21st–27th October 2011) and second (28th October–3rd November 2011) exposure periods

BTEXS	B		T		E		mX + pX		oX		S	
Period	I	II	I	II	I	II	I	II	I	II	I	II
*Cemetery in Opole*												
Average	1.38	4.15	2.83	13.2	<*MQL*	<*MQL*	1.58	1.95	<*MQL*	<*MQL*	<*MQL*	1.85
Median	1.30	4.15	2.70	13.2	<*MQL*	<*MQL*	1.60	2.00	<*MQL*	<*MQL*	<*MQL*	1.80
Min.	1.10	3.29	2.22	11.8	<*MQL*	<*MQL*	1.39	1.49	<*MQL*	<*MQL*	<*MQL*	1.52
Max	2.01	5.02	3.62	14.6	<*MQL*	<*MQL*	1.80	2.39	<*MQL*	<*MQL*	<*MQL*	2.23
SD	0.32	0.57	0.60	1.0	–	–	0.16	0.35	–	–	–	0.29
*Cemetery in Grodków*												
Average	1.43	3.50	2.97	13.1	<*MQL*	<*MQL*	2.25	2.25	<*MQL*	<*MQL*	<*MQL*	1.95
Median	1.35	3.30	2.95	12.9	<*MQL*	<*MQL*	2.25	2.05	<*MQL*	<*MQL*	<*MQL*	1.85
Min.	1.21	2.89	2.23	11.5	<*MQL*	<*MQL*	1.90	1.50	<*MQL*	<*MQL*	<*MQL*	1.50
Max	2.00	4.51	3.78	15.3	<*MQL*	<*MQL*	2.49	4.02	<*MQL*	<*MQL*	<*MQL*	2.49
SD	0.30	0.60	0.71	1.3	–	–	0.25	0.91	–	–	–	0.42

Average BTEXS concentration values determined for the blank samples were as follows: B < *MDL*, T = 2.2 μg m^−3^, E < *MDL*, Mx + pX = 0.41 μg m^−3^, oX < *MDL* and S < *MDL*.

The data presented in the table show significant increases in average concentrations of B (200 % and 144 % for the Opole and Grodków cemeteries, respectively) and T (366 % and 342 %) after air sampling during the festivities as compared to the period prior to the festival. The extended uncertainty in the measurements for methods for B and T was 17 % and 20 %, respectively (Table 1).

The results of the Wilcoxon test and *t* test, presented in Table 3, indicate statistically significant differences. During the first and second periods, E and oX concentrations were lower than the *MQL*. Also, S concentrations were lower than the *MQL* in the first period of exposure. Within the second period, S concentrations were higher than the *MQL*, but due to a lack of comparison with the first period of exposure, they could not be estimated in a quantitative manner; the differences between the concentrations of styrene and the MQL were within the expanded measurement uncertainty U (Table 1).

**Table 3:** α Values indicated by the Wilcoxon test and *t* test in order to present the statistically significant differences in B and T as well as in m + pX concentrations within periods I and II of air sampling at the cemeteries

Opole						Grodków					
Benzene		Toluene		mXylene + pXylene		Benzene		Toluene		mXylene + pXylene	
W test	*t* test	W test	*t* test	W test	*t* test	W test	*t* test	W test	*t* test	W test	*t* test
0.0350	1.13 × 10^−5^	0.0313	1.51 × 10^−6^	0.1010	3.18 × 10^−2^	0.0313	5.89 × 10^−4^	0.0355	2.23 × 10^−5^	0.4980	8.63 × 10^−2^

The average absolute B/T/S values in the air at the cemeteries during the festivities were: 3.83/13.2/1.9 μg m^−3^. No significant increases in the concentration of T were noticed within the first exposure period. Those concentrations were comparable with the value determined for the blank sample (2.2 μg m^−3^). Results of indications mX + pX within exposure periods I ad II (Table 2) differed from each other within the limits of a measure of uncertainty expressed by a SD. Moreover, in the case of mX + pX, the Wilcoxon test did not indicate statistically important differences between periods I and II. Also concentrations of mX + pX were comparable to concentrations from the city centres, to the effect that most probably these compounds are not emitted during candle burning.

Concentration values in the Opole and Grodków city centres, calculated according to formula no. 1 for E, oX and S, were lower than the *MQL*.

Some research on BTEXS emission and immission have focused on assessing benzene and toluene in air pollution, searching for relationships between T and B concentrations characteristic of various emission sources. For comparison, Table 4 presents results of the B and T concentration tests and the T/B values indicated during the autumn season in the air in urban and suburban areas in the state of Michigan, USA (Jia et al. 2008), in the industrial area of the city of Shizuoka, Japan (Ohura et al. 2006), near communication arteries in Antwerp, Belgium (Buczynska et al. 2009) as well as in the vicinity of the industrial part of A Coruña, Spain (Fernandez-Villarrenaga et al. 2004). The results of the year-long research carried out in the vicinity of communication arteries in Bari, Italy (Caselli et al. 2010) and in Zabrze, Poland (Pyta and Zajusz 2010) are also presented.

**Table 4:** A comparison of literature data regarding B and T concentrations (μg m^−3^) indicated in the ambient air

Research area	B	T	Τ/Β	References
Michigan (USA)	1.71	4.28	2.50	Jia et al. (2008)
Shizuoka (JAP)	0.94	6.4	6.81	Ohura et al. (2006)
Antwerpia (BEL)	2.5	9.5	3.80	Buczynska et al. (2009)
A Coruna (ESP)	3.43	23.6	6.88	Fernandez-Villarrenaga et al. (2004)
Bari (ITA)	3.01	6.21	2.06	Caselli et al. (2010)
Zabrze (POL)	2.84	4.06	1.43	Pyta and Zajusz (2010)

The data included in the table indicate that characteristic values of T/B factors for city and transport emission fall within the range of 2.06 to 3.80, whereas in the industrial areas they fall between 6.81 and 6.88. The industrial city of Zabrze (T/B = 1.43), where the hard coal mining and processing industry (coke plant, *i.a*.) is concentrated, appears to be an exception. The indicated average values of the T/B ratio in the cities and inthe cemeteries were within the ranges indicated by other authors for city and transport emission. The average benzene and toluene concentrations in Opole and Grodków were comparable and throughout the test period they amounted to 1.41 and 3.63 μg m^−3^, respectively. The value of T/B cemetery was comparable to T/B at designated traffic routes in Antwerp, Belgium (Table 4).

**Fig. 2:**
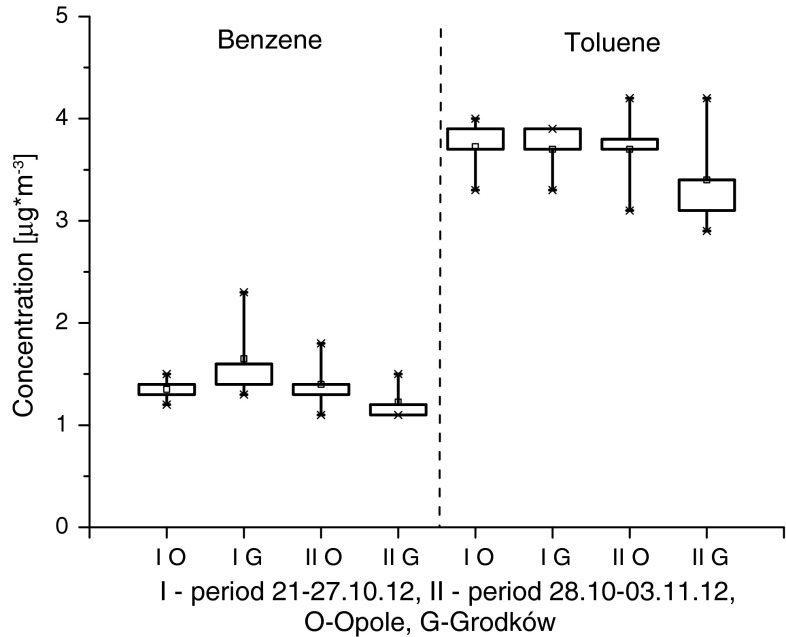
Benzene and toluene concentrations in the city centres of Opole and Grodków. *Boxes* show the range between the 25th and 75th percentiles. The *whiskers* extend from the edge of the *box* to the 5th and 95th percentiles of the data. The *squares* inside indicate median values

Figure 2 compares B and T concentrations in the city centres of Opole and Grodków. Maximum and minimum values, upper and lower quartiles, as well as medians have been indicated (McGill et al. 1978).

According to the data included in Table 2, during the first exposure period (prior to All Saints’ Day) B and T concentrations in the cemeteries amounted to 1.41 and 3.83 μg m^−3^, indicating that those values constitute a city background, statistically comparable for Opole and Grodków. Just to remind, the average benzene concentration values in the cemeteries in Opole and Grodków within the second exposure period (on All Saints’ Day) amounted to 2.83 and 2.97 μg m^−3^, and toluene concentrations amounted to 13.2 and 13.1 μg m^−3^, respectively. T/B ratios ran in the following manner: in the cities and in the first period of exposure at the cemetery: T/B_city_ = 2.65; while at the cemetery within the second period of exposure T/B_cemetery_ = 3.46.

During the seven-day exposure of the samplers in the cemeteries during the festival (28th October–3rd November), the average benzene concentration in the ambient air did not exceed acceptable limit values (5 μg m^−3^), with reference to the calendar year. Two-day exposure of the samplers demonstrated, however, an important rise in the benzene concentration during intensive burning of cemetery candles on November 1st and 2nd.

The average benzene concentration, calculated in accordance with formula no. 1, during 40 h of air sampling amounted to 14.9 μg m^−3^ at the cemetery in Opole and 12.3 μg m^−3^ at the cemetery in Grodków, i.e. it exceeded more than twice the acceptable concentration value with reference to the calendar year.

The test results indicate that as a result of candle and cemetery candle burning, BTEXS, mainly benzene, toluene and styrene, are emitted to the environment. Statistically significant differences in mX + pX concentrations were not noted. The average increase in the benzene concentration amounted to 200 % and 144 % at the cemeteries in Opole and Grodków, and the average rise in the toluene concentration amounted to 366 % and 342 %, respectively. The styrene concentration at the cemeteries during the second exposure period, including festivities, on average amounted to 1.9 μg m^−3^. During other periods and in other places, the styrene concentration was lower than the *MQL* (1.5 μg m^−3^). The average benzene and toluene concentrations in the cemeteries during the Festival (3.83 and 13.2 μg m^−3^, respectively) were higher than the average values indicated for many urban and industrial areas (Table 4, with the exception of A Coruña). A much greater rise in the concentrations of those analytes was observed within the third measurement period during intensive cemetery candle burning on 1st–2nd November. Then, the benzene concentration exceeded the permissible concentration value two fold, with reference to the calendar year (5 μg m^−3^).

The indicated average values of the T/B ratio in the cities and in the cemeteries were located within the range of factors established by other authors for city and transport emissions.
